# Integrating genetic and immune factors to uncover pathogenetic mechanisms of viral-associated pulmonary aspergillosis

**DOI:** 10.1128/mbio.01982-23

**Published:** 2024-04-23

**Authors:** Samuel M. Gonçalves, Inês Pereira, Simon Feys, Cristina Cunha, Georgios Chamilos, Martin Hoenigl, Joost Wauters, Frank L. van de Veerdonk, Agostinho Carvalho

**Affiliations:** 1 Life and Health Sciences Research Institute (ICVS), School of Medicine, University of Minho, Braga, Portugal; 2 ICVS/3B’s-PT Government Associate Laboratory, Guimarães/Braga, Portugal; 3 Medical Intensive Care Unit, Department of General Internal Medicine, University Hospitals Leuven, Leuven, Belgium; 4 Department of Microbiology, Immunology and Transplantation, KU Leuven, Leuven, Belgium; 5 Laboratory of Clinical Microbiology and Microbial Pathogenesis, School of Medicine, University of Crete, Heraklion, Crete, Greece; 6 Institute of Molecular Biology and Biotechnology, Foundation for Research and Technology, Heraklion, Crete, Greece; 7 Division of Infectious Diseases, ECMM Excellence Center for Medical Mycology, Department of Internal Medicine, Medical University of Graz, Graz, Austria; 8 BioTechMed, Graz, Austria; 9 Department of Internal Medicine, Radboud University Nijmegen Medical Centre, Nijmegen, the Netherlands; 10 Radboud Center for Infectious Diseases (RCI), Radboud University Nijmegen Medical Centre, Nijmegen, the Netherlands; Instituto Carlos Chagas, Curitiba, Brazil

**Keywords:** viral-associated pulmonary aspergillosis, innate immunity, host genetics, immunotherapy, influenza, COVID-19

## Abstract

Invasive pulmonary aspergillosis is a severe fungal infection primarily affecting immunocompromised patients. Individuals with severe viral infections have recently been identified as vulnerable to developing invasive fungal infections. Both influenza-associated pulmonary aspergillosis (IAPA) and COVID-19-associated pulmonary aspergillosis (CAPA) are linked to high mortality rates, emphasizing the urgent need for an improved understanding of disease pathogenesis to unveil new molecular targets with diagnostic and therapeutic potential. The recent establishment of animal models replicating the co-infection context has offered crucial insights into the mechanisms that underlie susceptibility to disease. However, the development and progression of human viral-fungal co-infections exhibit a significant degree of interindividual variability, even among patients with similar clinical conditions. This observation implies a significant role for host genetics, but information regarding the genetic basis for viral-fungal co-infections is currently limited. In this review, we discuss how genetic factors known to affect either antiviral or antifungal immunity could potentially reveal pathogenetic mechanisms that predispose to IAPA or CAPA and influence the overall disease course. These insights are anticipated to foster further research in both pre-clinical models and human patients, aiming to elucidate the complex pathophysiology of viral-associated pulmonary aspergillosis and contributing to the identification of new diagnostic and therapeutic targets to improve the management of these co-infections.

## INTRODUCTION

Recent progress in medical care has, paradoxically, contributed to an increased prevalence of severe fungal infections, notably invasive pulmonary aspergillosis (IPA) (1). IPA affects mostly hematological patients and recipients of solid-organ and stem-cell transplantation but has been increasingly diagnosed in patients with chronic lung diseases or those requiring intensive care due to severe viral infection (2–4). According to current estimates, more than 30 million people are at risk of IPA each year, with over 2 million patients developing it annually (5). Despite its global impact, characterized by high mortality rates, escalating healthcare costs, and the emergence of drug-resistant strains (6), diagnostic and therapeutic options for IPA remain limited. Recognizing the urgency, the World Health Organization recently designated *Aspergillus fumigatus* as one of the four “critical priority” pathogens in their inaugural fungal pathogen priority list, aimed at guiding research, development, and public health action (7).

Influenza-associated pulmonary aspergillosis (IAPA) was first documented in immunocompetent individuals, but it gained attention following the 2009 pandemic when influenza infection was identified as an independent risk factor for IPA in critically ill patients admitted to the intensive care unit (4). Similarly, in the recent COVID-19 pandemic, patients with severe acute respiratory syndrome coronavirus 2 (SARS-CoV-2) infection also showed increased susceptibility to fungal co-infections (2, 8, 9). Roughly 10%–20% of patients in the ICU are diagnosed with IAPA or COVID-19-associated pulmonary aspergillosis (CAPA), and these cases are linked to high mortality rates that exceed 50% (3, 10). Moreover, while the general incidence of severe COVID-19 has decreased during the vaccination era, a recent retrospective observational study reported a high burden of CAPA in severely immunocompromised patients requiring mechanical ventilation, with a major impact on outcome (11).

The development of viral-associated pulmonary aspergillosis (VAPA) entails complex tripartite interactions between the host, the fungus, and the virus (12). The host defense against inhaled fungal spores initiates in the anatomical barriers of the respiratory tract, with deposited conidia on the airway surface fluid being cleared by the ciliary action of the respiratory epithelium (13). It is noteworthy that both influenza and COVID-19 induce damage to the lung epithelium, characterized by disrupted epithelial junctions, impaired mucociliary clearance, and loss of cell functions (14, 15). The latter includes the suppression of the nicotinamide adenine dinucleotide phosphate oxidase (NADPH) complex and the release of antimicrobial proteins. Moreover, both infections trigger a severe inflammatory response, marked by the production of proinflammatory cytokines, such as interleukin-1β (IL-1β), IL-6, and tumor necrosis factor, commonly referred to as a “cytokine storm.” This heightened inflammatory response further contributes to the disruption of lung epithelial integrity. The damage inflicted by the influenza and SARS-CoV-2 viruses on the tracheobronchial epithelium might serve as a gateway for fungal entry, potentially making patients more prone to post-viral aspergillosis. This concept finds support in the observation of fungal growth in proximity to epithelial damage in co-infected patients (16).

Fungal infections, including VAPA, exhibit significant variability in their onset, progression, and outcome, even among patients with similar predisposing clinical conditions and environmental exposure. While virulence factors and mechanisms of adaptation of pathogens contribute to infection, genetic host factors also play a dominant role (17, 18). Our current understanding of the genetic basis of fungal infection has primarily derived from the study of patients with rare monogenic defects and from cohort-based investigations that identified common single nucleotide polymorphisms (SNPs) associated with infection, primarily in cohorts of immunocompromised individuals (19). However, knowledge about genetic susceptibility to VAPA and the possible molecular and cellular mechanisms involved is so far lacking. A comprehensive understanding of the genetic mechanisms governing antifungal immunity is anticipated to unlock unprecedented opportunities for personalized and more effective management of fungal infection (18). Viral-associated fungal infections, such as COVID-19-associated mucormycosis and candidemia, are extensively discussed in other reviews (3, 20, 21) and will not be addressed here.

This review explores recent advances in our understanding of the pathophysiological mechanisms influencing the development of VAPA. In particular, we discuss insights garnered from genetic studies conducted across various experimental and clinical models of IPA and their integration with immunological profiles reported in IAPA and CAPA. This will contribute to establishing a cohesive framework for understanding human susceptibility to VAPA, thereby revealing novel targets and pathways amenable to clinical intervention.

## GENETIC AND IMMUNE DETERMINANTS OF VIRAL AND FUNGAL RECOGNITION

**Fig 1:**
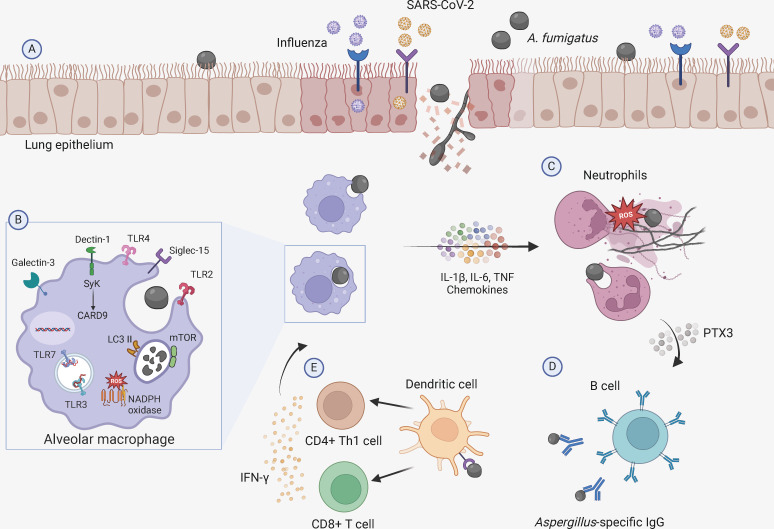
Immunological mechanisms of susceptibility to VAPA. (**A**) Viral infection promotes epithelial damage and dysregulated cell function, including desensitization of PRRs, suppression of the NADPH oxidase complex, and severe inflammatory responses, which may favor tissue invasion by *Aspergillus* and predispose to post-viral aspergillosis. (**B**) In the context of VAPA, alveolar macrophages recognize the fungal pathogen through various PRRs, including Toll-like receptors, dectin-1, galectin-3, and Siglec-15, and initiate intracellular signaling pathways that result in the activation of antifungal effector functions, including the induction of LC3-associated phagocytosis and the production of ROS, both mediated by NADPH oxidase complex, and the production of cytokines and chemokines. (**C**) Recruited neutrophils to the site of infection can phagocytose conidia, degranulate, release NETs, and produce ROS that contribute to fungal killing. (**D**) Soluble mediators from the humoral arm of innate immunity, such as PTX3 and SAP, also participate in the recognition of *Aspergillus* and regulation of antifungal responses. PTX3-producing neutrophils can stimulate B1a cells to generate innate antibodies capable of recognizing the fungus, which will then facilitate fungal recognition and elimination by innate cells. (**E**) Dendritic cells also play a pivotal role in recognizing and phagocytosing conidia, which leads to antigen presentation of fungus-derived peptides to T cells and the mounting of a protective adaptive antifungal response. Interferon-γ produced by Th1 cells primes macrophages and neutrophils to regulate antifungal immunity. The figure was created with BioRender (BioRender.com).

The damage inflicted by influenza and COVID-19 infections on the airway epithelium, coupled with the small size of the fungal spores, facilitates their bypassing of anatomical barriers and reaching the respiratory alveoli. Within the alveoli, conidia maturation will trigger morphological changes leading to the initial loss of the hydrophobin layer. Consequently, fungal pathogen-associated molecular patterns (PAMPs), such as β-1,3-glucan, mannan, chitin, and melanin, become exposed (22). An effective antifungal immune response relies largely on the selective recognition of these PAMPs by pattern recognition receptors (PRRs) expressed on phagocytes, including Toll-like receptors (TLRs) and C-type lectin receptors (CLRs) (Fig. 1).

Several studies have linked decreased expression levels of various PRRs following viral infection (23) and diminished responses to TLR ligands in influenza patients (24) to adverse outcomes in secondary bacterial infections. Of note, the downregulation of PRRs, attributable to loss-of-function variants in genes that encode these receptors, such as TLR3, has also been correlated with heightened disease severity and mortality in influenza (25) and increased susceptibility to IPA (26).

In a recent human retrospective observational study, transcriptome analysis and immune profiling performed on bronchoalveolar lavage fluid (BAL) samples obtained from patients admitted to the intensive care unit (27) diagnosed with IAPA or CAPA, compared to those with influenza or COVID-19 alone, respectively, revealed a downregulated expression of several genes encoding proteins with functions in fungal recognition (16). In particular, the expression of *TLR2* was markedly downregulated in BAL samples from patients with IAPA. These findings suggest that impaired TLR2 activation and downstream signaling may contribute to an increased susceptibility to superinfection. In support of this, the rs5743708 SNP in *TLR2*, leading to the amino acid change R753Q that disrupts TLR2 signaling by impairing its tyrosine phosphorylation, dimerization with TLR6, and recruitment of Mal and MyD88 (28), was previously associated with predisposition to invasive fungal disease in patients with acute myeloid leukemia (29).

Genetic variation in TLR3, a recognized receptor for viral double-stranded RNA, has also been linked to an increased incidence of influenza-related pneumonia (30) and associated mortality (25). Because the activation of TLR3-dependent pathways in both epithelial cells and dendritic cells (DCs) has been shown to confer resistance to aspergillosis (26, 31), TLR3 was also suggested to participate in the recognition of fungal nucleic acids. This notion gained support from the association of the rs3775296 SNP in *TLR3* with the risk of IPA in recipients of stem-cell transplantation (26). Mechanistically, the decreased expression of *TLR3* in DCs from variant carriers and the ensuing impaired detection of fungal RNA resulted in the defective priming of memory CD8^+^ T cell responses, rendering individuals more susceptible to infection. This same variant was incorporated into a polygenic risk score for predicting cytomegalovirus reactivation after transplantation (25), suggesting a potential dual effect of the SNP on the recognition of both viral and fungal pathogens, with possible consequences for the development of fungal superinfections, particularly IAPA.

However, not all genetic variants associated with a poor prognosis in viral-infected patients may necessarily contribute to a secondary fungal infection, as exemplified by the case of TLR7. Genetic variants in *TLR7* that lead to the transcriptional downregulation of type I interferon (IFN) signaling have been identified to predispose young males to severe COVID-19 (32). Nevertheless, TLR7 has been shown to exacerbate the development of IPA in mouse models by hindering phagocytosis and fungal killing by macrophages (33). This suggests that, despite its importance in COVID-19, TLR7 signaling may play a relatively minor role in protecting against CAPA.

Besides the classical nucleic acid-recognizing TLRs, TLR4 has also been demonstrated to protect against influenza (34). Moreover, although the fungal ligands recognized by TLR4 remain unknown, a haplotype consisting of the coding variants rs4986790 (D299G) and rs2986791 (T399I) in *TLR4* has been associated with an increased risk of IPA in stem-cell transplant recipients (35) and immunocompetent individuals suffering from chronic pulmonary aspergillosis (36). It remains, however, to be assessed whether genetic variation affecting TLR4 expression and/or function might also represent an important susceptibility factor to VAPA.

The activation of antifungal immune responses primarily depends on CLRs, which are pivotal in recognizing fungal glucans, glycolipids, and glycoproteins (22). In a transcriptomic data set derived from BAL samples of IAPA patients (16), the differential expression of several genes related to the recognition of glycan moieties abundant in the fungal cell wall, such as *CLEC7A* (dectin-1), *SIGLEC15*, and *LGALS3* (galectin-3), emerged as predictors of mortality of ICU patients diagnosed with IAPA (37).

The significance of dectin-1, the primary receptor for β-1,3-glucan, in IPA, has been established through the identification of the stop codon SNP Y238X and its association with an increased risk of IPA in both stem-cell transplant recipients and hematological patients (38, 39). The Y238X SNP results in the truncation of dectin-1 at the carbohydrate recognition domain, leading to a reduced surface expression of the receptor and, consequently, a defective production of cytokines by myeloid cells. Importantly, this association was observed regardless of whether the SNP was present in the donor or patient genomes. This association model was further confirmed in an independent genetic association study involving transplant recipients and respective donors (40). These findings suggest that dectin-1 may play a relevant role that extends beyond the involvement of hematopoietic cells in contributing to antifungal immune responses.

Recognition of β-1,3-glucan through dectin-1 has been demonstrated to trigger trained immunity by influencing specific pathways of cellular metabolism (41, 42). Moreover, the activation of PRRs on antigen-presenting cells during a viral infection induces an epigenetic functional reprogramming that alters their responsiveness to subsequent challenges (43). Along these lines, an *ex vivo* infection model involving human alveolar macrophages showed a significant decrease in *CLEC7A* expression following influenza infection (44). It is thus plausible that the Y238X SNP and other dectin-1 variants may predispose individuals to IAPA by impairing both virus-induced trained immunity and the “natural” trained immunity that arises from constant exposure to fungi.

Dectin-1 activates diverse cellular responses through the spleen tyrosine kinase (Syk)/caspase recruitment domain-containing protein 9 (CARD9) signaling pathway (22). Rare mutations in *CARD9* have been linked to extrapulmonary invasive aspergillosis as the result of an impaired accumulation of neutrophils in infected tissues (45). Notably, the loss of CARD9-mediated innate activation attenuated severe influenza pneumonia without compromising host viral immunity (46). This observation suggests that the impact of genetic variation in *CARD9* on IAPA, if any, is specifically directed on antifungal rather than antiviral immunity.

In addition to *CLEC7A*, reduced expression of the *SIGLEC15* and *LGALS3* genes has also been identified as predictors of mortality following IAPA (37). Siglec-15 is a sialic acid-binding receptor expressed on the surface of immune cells (47). In line with its ability to bind sialic acids in the conidial surface, *SIGLEC15* expression was upregulated in mononuclear cells after fungal stimulation, and it was required for effective fungicidal activity (48). Furthermore, the rs2919643 SNP in *SIGLEC15* (F273I) has been associated with reduced production of cytokines, including IL-1β and IFN-γ, in response to fungal stimulation. These findings highlight the potential significance of Siglec-15 in the context of antifungal immunity and emphasize the need for further investigation to validate its role as a genetic determinant in susceptibility to IAPA.

The use of neuraminidase inhibitors, such as oseltamivir, which cleave sialic acid residues following influenza infection might therefore influence antifungal responses and susceptibility to IAPA. In this regard, oseltamivir treatment has been shown to increase susceptibility to aspergillosis in corticosteroid-treated mouse models through mechanisms that compromise the effector functions of immune cells (48). However, in a viral-fungal co-infection model, early administration of oseltamivir prevented severe influenza pneumonia and mitigated the development of IAPA and associated mortality (49). This suggests that the timing of administration of oseltamivir is important: early treatment may mitigate severe influenza, thereby reducing the risk for IAPA, but if initiated when severe infection is already established, it could be detrimental by facilitating IAPA.

The *LGALS3* gene encodes galectin-3, a beta-galactoside-binding lectin proposed to function not only as a PRR but also as a danger-associated molecular pattern (DAMP) (50). Besides its role in the immune response, galectin-3 can interact with glycans on the viral surface, facilitating their entry into host cells and promoting infection. While galectin-3 expression was significantly upregulated in mice and humans with IPA (51), *LGALS3* transcript levels were instead lower in BAL from patients with IAPA compared to those with influenza alone (16). This observation suggests that viral infection likely conditions the expression and/or function of galectin-3, potentially dampening its antifungal activity. Indeed, mice lacking galectin-3 exhibited increased fungal burden and mortality in experimental aspergillosis compared to wild-type mice (51). This outcome was attributed to impaired neutrophil recruitment to the site of infection, although the total number of lung neutrophils remained unaltered. Whether these mice are more susceptible in viral-fungal co-infection models and if genetic variation in *LGALS3* could account for differences in the development of IAPA and its associated mortality remains to be evaluated.

PRRs can also respond to products released from damaged host cells. In the lungs, virus-infected cells release DAMPs, which neighboring epithelial cells and resident alveolar macrophages detect through PRRs. In this context, the receptor for advanced glycation end products (RAGE) binds to various extracellular DAMPs, including HMGB1, S100 proteins, and modified lipids, proteins, and nucleic acids released from damaged or dying cells. Importantly, RAGE was reported to be detrimental during influenza pneumonia by hindering viral clearance and suppressing cellular T cell responses and the activation of neutrophils (52). This receptor has also been implicated in the regulation of inflammation during fungal disease (53). Given that genetic variants leading to the hyperfunction of the DAMP/RAGE axis were implicated in susceptibility to IPA in transplant patients (54), the amplification of DAMP-mediated signals released by viral-damaged cells could establish permissive inflammatory conditions that further predispose individuals to secondary fungal infections.

## NATURAL AND HUMORAL IMMUNITY TO VIRAL-ASSOCIATED ASPERGILLOSIS

The interaction between fungi and phagocytes involves various soluble mediators, including pentraxins, complement proteins, ficolins, and collectins—molecules within the humoral arm of the innate immune system (55). Of note, patients with IPA exhibited low circulating concentrations of mannose-binding lectin (MBL), an innate immune lectin that binds different glycans (56). In contrast, a glycomic analysis of the host response to influenza infection identified high mannose as a key mediator of disease severity (57), and SNPs in *MBL2* were associated with the severity of COVID-19 (58). Considering that the expression levels, functional activity, or both of MBL are widely regulated at the genetic level (59), and given its implications for both viral and fungal infections, it is conceivable that SNPs in *MBL2* also represent significant candidates for susceptibility to VAPA.

Pentraxins form an evolutionarily conserved family of acute-phase serum proteins that exhibit pattern recognition abilities toward microbial moieties from a diverse array of microorganisms (55). The long pentraxin PTX3 plays a crucial role in binding to *A. fumigatus*, facilitating its recognition by phagocytes, including neutrophils and alveolar macrophages. The essential role of PTX3 in host antifungal defense was established *in vivo* through the heightened susceptibility of mice lacking PTX3 to experimental aspergillosis and the observation that administration of exogenous PTX3 rescued antifungal effector functions and restored survival (60).

In humans, the pivotal role of PTX3 became evident by the increased susceptibility to IPA of stem-cell transplant patients who carried specific donor SNPs that affected PTX3 expression (61). This association was not only confirmed in a large, independent study (40) but also extended across different clinical settings, encompassing solid organ transplant recipients (62, 63), acute myeloid leukemia patients (64), and patients with chronic obstructive pulmonary disease (65). Mechanistically, neutrophils from PTX3-deficient donors exhibited a defective capacity to phagocytose and eliminate fungal spores, a phenotype further supported by the lack of association with IPA in patients with severe neutropenia (61, 64). Importantly, the treatment of neutrophils with recombinant PTX3 restored both their phagocytosis and fungicidal activity (61).

The levels of PTX3 have been reported to exhibit a significant increase in patients with severe COVID-19 (66) and in the lungs of mice infected with influenza (67). While PTX3 was also elevated in BAL samples from patients with IPA (61), it did not differ significantly between IAPA or CAPA and patients with viral infection alone (16). Nonetheless, it is possible that genetic variants in *PTX3*, known to regulate its expression in response to infection, could contribute to host susceptibility to VAPA. Moreover, genetic variation in other pentraxins that also influence the effector functions of immune cells in antifungal immunity, such as serum amyloid P (SAP) (68, 69), might also represent important elements of susceptibility to VAPA. It is noteworthy that, besides a crucial role in IPA, SAP has been identified as a sialylated glycoprotein inhibitor of influenza viruses (70). Therefore, considering the seemingly overlapping roles of these pentraxins in antifungal defense, investigating their potential involvement in VAPA is warranted.

Natural IgG antibodies produced by B1a cells play an essential role in host resistance against viral- and steroid-associated aspergillosis by promoting neutrophil-mediated phagocytosis (71). Influenza infection has been shown to induce the death of B1a cells, leading to impaired production of natural antibodies and a weakened ability of neutrophils to capture the fungus. Similarly, patients with influenza infection or COVID-19 exhibit decreased levels of anti-*Aspergillus* IgG antibodies compared to healthy individuals, likely due to the decreased circulating B1 cells in these patients (71). Of significant note, the development of severe COVID-19 appears to be more frequent among patients undergoing B-cell depletion therapy, resulting in impaired humoral immune responses and hyperactivation of T-cells (72).

Neutrophils are known to produce PTX3, which can, in turn, stimulate splenic marginal zone B cells to generate innate antibodies capable of recognizing encapsulated bacteria through T-cell-independent processes (73). Given that *PTX3* SNPs affect the regulatory role of neutrophils in B-cell function, including class switching, plasmablast expansion, and antibody production (73), it is conceivable that these genetic variants might compromise the B1a–natural IgG antibody–neutrophil axis and facilitate fungal dissemination during co-infections. Although clinical data on this aspect are currently limited, these observations suggest the potential utility of PTX3 in novel therapeutic strategies aimed at treating or preventing VAPA through mechanisms involving innate humoral immunity (74).

## DEFECTS IN ANTI-MICROBIAL EFFECTOR FUNCTIONS OF IMMUNE CELLS

Similar to other forms of invasive aspergillosis, effective antifungal defense in VAPA primarily involves alveolar macrophages and neutrophils (3, 75). Influenza infection leads to a depletion of alveolar macrophages in mice, increasing susceptibility to secondary bacterial infections (76). Alveolar macrophages are depleted as well in the lungs of patients with severe COVID-19 and are replaced by hyperinflammatory monocytes, which do not fully differentiate to well-functioning macrophages (77). Nevertheless, a downregulation of numerous molecules with roles in the antifungal effector functions of macrophages was observed (16). One of these downregulated molecules was C-X-C motif chemokine ligand 10 (CXCL10), a chemokine that participates in the recruitment of neutrophils. Of note, SNPs in *CXCL10* were associated with the risk for IPA (78), an effect attributed to a decreased *CXCL10* expression by DCs from carriers. Moreover, survivors of IPA displayed higher CXCL10 levels compared to non-infected patients. Whether these SNPs, by affecting the expression of *CXCL10* and restraining neutrophil recruitment, could contribute to the development of IAPA or CAPA remains to be explored.

Regardless of the mechanisms involved, the role of neutrophil recruitment in IAPA remains controversial. While decreased neutrophil recruitment has been found to exacerbate susceptibility to fungal infection in mouse models of IAPA (79, 80), others have not observed this in either mice (81) or humans (71). These disparate results suggest that a deficiency in neutrophil recruitment *per se* may not fully explain the acquired susceptibility to IAPA and that additional defects in antifungal effector mechanisms may also play a leading role.

One of the key mechanisms in antifungal host defense is the production of reactive oxygen species (ROS) by macrophages and neutrophils, mediated through the NADPH oxidase complex (75). Despite evidence suggesting that the inhibition of ROS production can ameliorate influenza-induced lung injury (82), influenza infection has been shown to suppress NADPH oxidase-dependent ROS production in alveolar macrophages and neutrophils (83). This phenotype impacts the bactericidal capacity of these cells and results in increased susceptibility to secondary bacterial infection.

The crucial role of the NADPH oxidase complex is emphasized by the remarkable susceptibility to IPA in patients with chronic granulomatous disease (CGD), a condition characterized by genetic deficiency in components of the NADPH oxidase complex. Furthermore, the impaired production of ROS in CGD patients results in a defective induction of a noncanonical form of autophagy known as LC3-associated phagocytosis (LAP) (84). LAP is recognized as a critical process for fungal elimination and is targeted by fungal melanin to enhance pathogenicity (85).

Given its central role in antifungal immunity, genetic variation in molecular players that regulate LAP activation has been reported to influence susceptibility to IPA. The rs12885713 SNP in the core promoter of the calmodulin-1 gene (*CALM1*) was reported to increase the risk of IPA in transplant recipients (86). This association was corroborated by the requirement for calcium signals and calmodulin recruitment to the phagosome in regulating various molecular components of LAP, including Rubicon and the NADPH oxidase. Similarly, increased susceptibility to IPA was also disclosed among carriers of SNPs in molecular regulators of the assembly of functional phagolysosomes against fungal infection (87) and in specific host molecules that the fungus manipulates to promote phagosomal escape (88). Supporting a role for phagosome biogenesis and LAP in VAPA, the expression of genes involved in LAP, namely *MAP1LC3B* (microtubule-associated protein 1 light chain 3 beta) and *SQSTM1* (sequestosome-1), was decreased in IAPA and CAPA compared to patients with viral infection alone (16). In contrast, the gene encoding for the CDC20 protein involved in LC3 degradation was upregulated in the co-infected patients. These results suggest that genetic variants impairing LAP activation may predispose individuals to VAPA.

In a recent study, the impact of genome-wide SNPs on ROS production in response to fungal infection was evaluated in a population-based cohort of Western European ancestry (89). The study identified several genetic loci that regulate ROS levels, referred to as ROS quantitative trait loci, among which the rs4685368 SNP was identified as a major risk factor for IPA in transplant patients, an association supported by the impaired production of ROS after fungal stimulation by cells from carriers of the risk genotype. These findings suggest that genetic-driven suppression of ROS could not only impair their direct antifungal activity but also compromise the activation of LAP. It is also noteworthy that LAP is dependent on the activation of dectin-1/Syk kinase/NADPH oxidase signaling (90), establishing a link between pattern recognition by CLRs and NADPH oxidase-mediated host defense. The decreased expression of dectin-1 and its association with increased mortality of IAPA patients (37) could therefore also result from defects in LAP activation.

The role of ROS-mediated release of neutrophil extracellular traps (NETs) in fungal elimination, known as NETosis, is a subject of debate in the field (91, 92). The prevailing concept suggests that NETs may exert a fungistatic effect rather than directly killing fungi, aiming to prevent further spreading of the infection. Importantly, PTX3 is stored in neutrophil granules and is found within NETs (93), highlighting a potential involvement of PTX3 SNPs in both NET formation and their effectiveness against fungal infection. The formation of NETs is a common process observed in the lungs of severe COVID-19 patients (94), indicating a potential pathogenetic role of NETs during viral infection. The use of single-cell RNA sequencing (scRNA-seq) of BAL samples revealed that patients with CAPA exhibited significantly lower neutrophil fractions in BAL compared to patients with COVID-19 alone, caused at least in part by increased NETosis in the co-infected patients (95). Importantly, pronounced NETosis was associated with lower mortality rates in CAPA patients. Further investigation on the associations between NETosis and outcome and their link to altered ROS production, and whether these are also present in IAPA, is warranted. Therefore, in contrast to its detrimental role in severe COVID-19, CAPA patients appear to benefit from NETosis to effectively counter fungal infection and dissemination.

Another crucial process involved in regulating the antifungal effector functions of immune cells involves their capacity to reprogram cellular metabolism (96). In the context of aspergillosis, macrophages depend on enhanced glucose metabolism, driven by the activation of mammalian target of rapamycin (mTOR) and hypoxia-inducible factor-1α (HIF-1α), to support their antifungal effector activity (97). Notably, scRNA-seq conducted on BAL samples indicated a downregulation of glycolysis-related genes, including mTOR signaling, alongside an upregulation of oxidative phosphorylation in monocytes and macrophages from CAPA patients compared to those with COVID-19 alone (95). In addition, reduced expression of the *HIF1A* gene was also observed in patients with IAPA and CAPA (16). It is noteworthy that long-term metabolic dysregulation has been demonstrated to impact disease progression and immune responses in influenza (98) and COVID-19 (99), potentially influencing susceptibility to secondary fungal infections.

Genetic variation in essential mechanisms of cellular metabolism could also play a significant role. Specifically, SNPs in the *PFKFB3* gene encoding 6-phosphofructo-2-kinase/fructose-2,6-biphosphatase 3, a critical regulator of glucose metabolism, have been found to impair macrophage effector functions and predispose transplant recipients to IPA (100). Moreover, the ability of neutrophils to eliminate fungi relies on their capacity to upregulate glucose uptake via the selective expression of glucose transporter 1 (GLUT1) (101). It is noteworthy that the release of NETs by neutrophils is also contingent on the increase of cell membrane GLUT1 and glucose uptake (102). Therefore, a defective ability to reprogram cellular metabolism as a consequence of viral infection and/or the presence of SNPs in essential metabolic regulators may hinder NETosis and contribute to the development and outcome of VAPA.

Genetic variation affecting adaptive immune responses to fungi may also be relevant in the context of VAPA. For example, loss-of-function mutations in STAT3 are linked to autosomal dominant hyper-immunoglobulin E (IgE) syndrome (103), which underlies a heightened susceptibility to IPA (104). Patients with STAT3 deficiency exhibit decreased production of IFN-γ, IL-17, and IL-22, leading to a flawed adaptive immune response against *Aspergillus* infection (105). Furthermore, impaired Th17 responses are observed during influenza infection and are correlated with reduced neutrophil recruitment. This suggests that functional defects in the Th17 pathway among viral-infected patients, potentially caused by common variants affecting STAT3 function or other signaling pathways such as JAK/STAT (106), could also play a role in VAPA.

## IMMUNOMODULATION IN VIRAL-ASSOCIATED ASPERGILLOSIS: FRIEND OR FOE?

To address the hyperinflammatory state associated with severe viral infection, the predominant treatment options involve immunomodulatory drugs, such as corticosteroids and cytokine blockers, in particular, in severe COVID-19 (107, 108). In severe influenza, anti-inflammatory agents must be used with caution, and several trials have suggested increased mortality in patients who receive corticosteroids early during ICU stay (109). While they are often administered to patients with severe viral pneumonia, these therapies suppress the activation of both innate and adaptive antimicrobial responses and represent, therefore, important contributors to secondary fungal infections. Corticosteroids exert a suppressive effect on various immune cells, including neutrophils and monocytes/macrophages, and also T cells. Consequently, the administration of high-dose systemic corticosteroids poses a significant risk factor for the development of aspergillosis (110). Notably, the use of corticosteroids has been demonstrated to restrain the phagosomal recruitment of the LC3 protein, hampering the activation of LAP and fungal elimination (90).

The blockade of IL-6 with tocilizumab has proven to be effective in the treatment of severe COVID-19 by reversing hyperinflammation and preventing subsequent tissue damage (107). However, the use of tocilizumab has been linked to impaired effector functions of phagocytes and suppressed Th17 responses, increasing the vulnerability to secondary fungal infections (111). A similar profile of immune dysfunction was observed in a study where genetic variants near the *IL6* locus were shown to contribute to blastomycosis (112). Therefore, and although genetic variation in *IL6* has not been directly associated with IPA, these observations imply its potential involvement in the development of VAPA.

Severe viral infections often trigger the activation of inflammasomes, leading to the release of the proinflammatory cytokine IL-1β. Interestingly, a blunted transcriptional response to IL-1β was exhibited by patients with IAPA compared to those with influenza alone (16). This suggests that, despite the general increase of IL-1β during viral infection, its expression is suppressed in the context of IAPA. The specific mechanisms involved remain unknown, but there appears to be a role played by the fungal infection itself, as genetic variants that reduce the production of IL-1β have been shown to contribute to susceptibility to invasive fungal infections following solid-organ transplantation (113). Likewise, loss-of-function SNPs affecting the NLRP3 inflammasome have been associated with the development of IPA in non-neutropenic patients (114).

A recent phase III clinical trial demonstrated that early treatment of COVID-19 with the IL-1 receptor antagonist anakinra leads to reduced mortality and shorter hospital stays (115). Whether this approach could prevent co-infections or, conversely, increase susceptibility to them remains a subject for further investigation. However, it is noteworthy that IL-1 receptor blockade has been shown to alleviate inflammation and limit susceptibility to experimental aspergillosis in conditions such as CGD (84) and cystic fibrosis (116).

**Fig 2:**
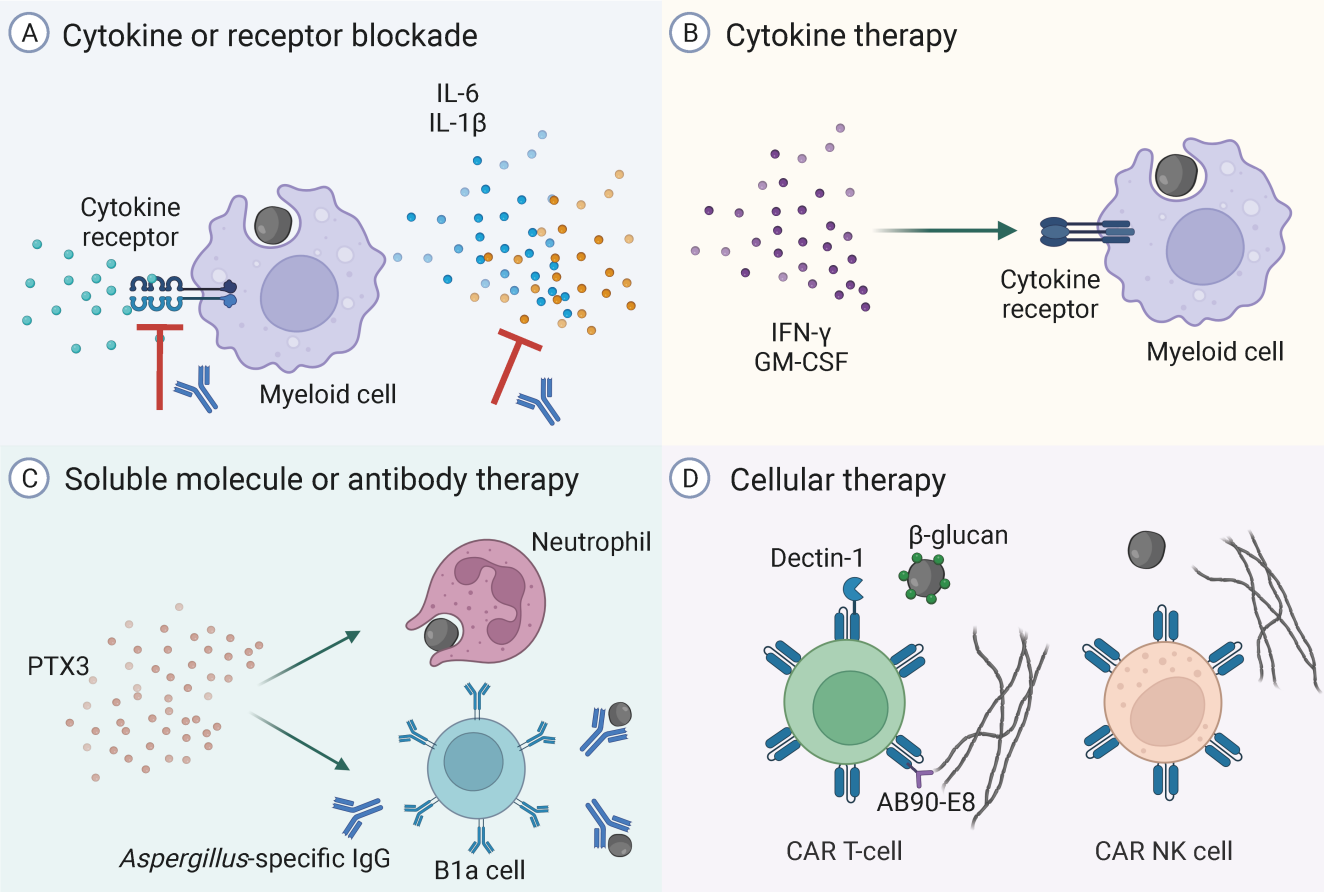
Host-directed therapies to treat or prevent VAPA. (**A**) The blockade of cytokines or their receptors reduces the damage to the lung epithelium caused by the severe hyperinflammation associated with the viral infection and could improve the antifungal effector activity of myeloid cells. (**B**) Administration of recombinant cytokines, such as IFN-γ or GM-CSF, could be used to overcome selected defects in signaling pathways and the functional activity of myeloid cells. (**C**) Therapy with soluble humoral mediators, such as PTX3, could act directly on neutrophils to enhance their phagocytic and fungicidal capacity and, in parallel, promote the secretion of fungus-specific natural antibodies by B1a cells. These fungus-specific innate antibodies could also be administered directly to tackle humoral defects and the B-cell suppression common in severe viral infections. (**D**) Chimeric antigen receptor (CAR) T-cells or CAR NK cells engineered for improved fungal recognition, e.g., through the expression of PRRs such as dectin-1 or recognition domains (AB90-E8) that target conserved fungal antigens in the fungal cell wall, have the capacity to promote direct antifungal effects. The figure was created with BioRender (BioRender.com).

It is now clear that, in addition to antifungal treatment, the optimal management of viral-associated pulmonary aspergillosis should ideally incorporate adjunctive host-directed therapies (Fig. 2) aimed at resolving immunopathology, while ensuring a balanced immune response that is essential for effective fungal elimination. Although potential targets, such as cytokines and their receptors, have been suggested, the effectiveness of these approaches requires a profound understanding of the context- and concentration-dependent biological effects of these molecules.

Common genetic variation is known to influence the biological levels of cytokines and the activity of their receptors. Specifically, the rs1800896 SNP, located in the promoter region of the gene encoding the immunoregulatory cytokine IL-10, has been found to promote increased levels of secreted IL-10 following fungal infection *in vitro* (117). This elevated production of IL-10 leads to an overall skew toward anti-inflammatory responses and compromised fungicidal activity, thereby predisposing transplant recipients to IPA. Of relevance, increased levels of IL-10 have been recognized as an immunological hallmark observed in patients with severe influenza, and these have been correlated with disease progression and suggested to contribute to secondary opportunistic infections (118).

Other cytokines have recently gained more attention in the context of host-directed medicine in invasive fungal infections. Two recent case series reported a potentially beneficial effect of IFN-γ in treating immune paralysis in patients with severe COVID-19 (119, 120). In the context of fungal infection, IFN-γ has also been shown to restore immune function in patients with fungal sepsis, including antigen presentation and the capacity of immune cells to produce proinflammatory cytokines (121). Along the same line, the promoter rs2069705 SNP in the *IFNG* gene, resulting in increased cytokine production, was found to confer resistance to IPA through a mechanism that involved the enhanced fungicidal activity of macrophages (122). Whether this might be related to the ability of IFN-γ to promote the direct activation of LAP and fungal elimination (123) remains to be assessed. Whatever the mechanism, a downregulation of IFN-γ signaling was observed in patients with IAPA and CAPA compared to those with the viral infection alone (16), suggesting the potential usefulness of immunotherapy with IFN-γ in the context of VAPA.

## TRANSLATING FUNDAMENTAL KNOWLEDGE INTO CLINICAL APPLICATION

Translating knowledge about the genetic mechanisms governing antifungal immunity and susceptibility to fungal infections into the clinical management of viral-associated pulmonary aspergillosis holds the potential to address critical unmet needs in clinical disease management. This includes facilitating earlier diagnosis of invasive aspergillosis or at the least the identification of the patients at the highest risk, followed by early treatment with enhanced efficacy achieved by the combination of antifungal therapy with immunotherapy targeting the host response. Moreover, genetic risk factors could also be used to guide antifungal prophylaxis in severe influenza/COVID-19 patients without fungal infection at ICU admission.

Reducing the devastating mortality rates associated with IAPA and CAPA remains a significant unmet challenge in clinical management. While IAPA is often present at ICU admission, CAPA is typically diagnosed after 6–10 days (2, 3, 124), providing a window for earlier diagnosis and intervention. The elevated mortality rates linked to CAPA are even higher when diagnosis is delayed. Mortality was 61% in cases with positive BAL galactomannan and culture (125), 76% in those with three or more positive mycological test results (27), and ranged from 87% to 100% in cases with positive serum galactomannan or plasma PCR test, but also in those with positive direct examination from BAL (27, 126). Initiating early treatment at the first positive test result and before (multiple) tests become positive may therefore improve survival. However, currently available tests lack the ability to distinguish between invasive infection and colonization. Combining existing detection tests, such as galactomannan, with host-derived biomarkers or cytokines could overcome this limitation and mitigate the risk of unnecessary overtreatment.

To enable even earlier initiation of antifungal treatment, host-derived biomarkers indicating lower levels of selected transcripts from myeloid innate immunity or downregulation of IFN-γ signaling (16) have the potential to assist clinicians in pinpointing individuals at highest risk who may benefit not only from prompt and aggressive diagnostics but also the initiation of empirical treatment for VAPA at the time of clinical deterioration and before diagnostic test results become available. The same holds true for a better definition of the patient population that could benefit the most from antifungal prophylaxis, for which single-center studies have suggested potential advantages in reducing the incidence of CAPA (127, 128) and IAPA (129), but have failed to demonstrate a significant impact on overall mortality.

Immunotherapy targeting fundamental immunological mechanisms that contribute to the development of IAPA and CAPA holds promise as an adjunctive treatment or prophylactic measure. These approaches aim to enhance the antifungal host response and improve fungal clearance (130). It is crucial to note that findings from immunotherapy studies conducted in patients with classical risk factors for IPA or mouse models resembling those conditions may not directly apply to VAPA patients. This distinction is important due to the hyperinflammatory environment in the lungs and the absence of classical risk factors in viral-fungal co-infections. Among the most promising approaches for VAPA are (i) recombinant interferon-gamma (rIFN-γ), currently employed as prophylaxis in patients with chronic granulomatous disease (131), which could help overcome the impaired IFN-γ signaling identified in VAPA, and (ii) natural anti-*Aspergillus* antibodies or PTX3 for tackling IgG deficiency and B-cell suppression in patients with severe influenza or COVID-19 (71, 132, 133).

In conclusion, the recent advances in our understanding of the genetic and immune factors driving viral-associated pulmonary aspergillosis offer the prospect of personalized management for these severe superinfections. However, the rapid progression of IAPA and CAPA in some patients demands treatments within hours to allow maximum chance of survival. This urgency may restrict the practicality of personalized treatment strategies to those that rely on genetic and immunological markers that are rapidly available to clinicians. For such markers to be effective, they should have a short turnaround time, require minimal consent and paperwork—especially those implemented in ICU settings—and be cost-effective. Developing these markers as rapid point-of-care tests could meet these requirements.

Despite the ongoing development of new antifungals, it is essential to also emphasize the role of the host and the potential of immunomodulation in improving VAPA outcomes. To comprehensively explore the potential of genetic and immune markers for antifungal treatment stratification and assess the added benefits of immunomodulation, there is a pressing need for high-quality, preferably randomized, and multicenter clinical trials. These trials are crucial to establishing routine, evidence-based practices for personalized management pathways and incorporating immunomodulation as an adjunct therapy in VAPA.
